# Predictive Value of miR-146a rs2431697 Polymorphism to Myelofibrosis Progression in Patients with Myeloproliferative Neoplasm

**DOI:** 10.31557/APJCP.2021.22.11.3585

**Published:** 2021-11

**Authors:** Salah Aref, Doaa Atia, Ahmed Al Tantawy, Mohamed Al Boghdady, Enas Gouda

**Affiliations:** 1 *Hematology Unit, Department of Clinical Pathology, Faculty of Medicine, Mansoura University, Egypt.*; 2 *Medical Oncology Unit, Mansoura University Oncology Center (MUOC) Mansoura University; Egypt.*; 3 *Hematology Unit, Mansoura University Oncology Center (MUOC), Mansoura University, Egypt. *

**Keywords:** miR-146a, IL-1β- NF-κB, NLRP3, MPN, Myelofibrosis

## Abstract

**Background:**

Bone marrow myelofibrosis (BMF) that develop on top of Polycythaemia vera (PV) and essential thrombocythemia leads to shortening of the patient’s overall survival. This study aimed to address the impact of miR-146a rs2431697 polymorphism on inflammatory biomarkers and genes expression and the hazards of myelofibrosis progression.

**Patients and Methods:**

The study included 88 myeloproliferative neoplasm (40 PV; 27 ET; 21 MF) and 90 healthy controls. For all investigated subjects miR-146a rs2431697 genotypes were identified by sequencing and the expression of miR-146a; IL-1β; NF-κB; a NOD-like receptor family, pyrin domain containing 3 (NLRP3) genes were estimated by real time PCR.

**Results:**

miR146a genotypes revealed that there was significant association between TT and TC genotypes with MF. The degree of miR146a expression was significantly reduced in MF as compared to both PV and ET. In contrast; the levels of IL-1β; NF-κB; NLRP3 genes expression were significantly elevated in MF patients group as compared to PV and ET patients’ group. Multivariate analysis identified TT genotype as poor predictor of MF progression.

**Conclusion:**

miR-146a rs2431697 TT genotype is associated with high risk of MF progression in MPN patients. Targeting of IL-1β; NF-κB; NLRP3 genes might help in hindering of MF progression in MPN patients,

## Introduction

The Philadelphia negative Myeloproliferative neoplasms (MPNs) (BCR-ABL-ve MPNs) are the most frequent diseases among the myeloproliferative disorders. MPNs are subcategorized into 3 subtypes that include polycythemia Vera (PV), essential thrombocytosis (ET), and Myelofibrosis (MF). MPN is characterized by progressive production of mature functioning terminal cells (Red cells and Platelets). During the progressive course of PV and ET; some of the patients transformed into MF which usually associated with complications namely thrombosis, hemorrhages, and worse complication which is transformation to acute myeloid leukemia (AML) (Vainchenker and Kralovics, 2017). 

The discovery of driver mutations namely JAK2, CALR, and MPL reveal that activated JAK-STAT signaling is main trigger of MF, supporting a rationale for JAK inhibition. However, JAK inhibition alone is insufficient for long-term remission and offers modest, if any, disease-modifying effects (Zhou et al., 2020). 

It is become clear that there is a close link between chronic inflammation and MPN pathogenesis. The expression of IL-1 which is an inflammatory cytokine was found to be increased in MPN. Also, the incidence of MPN patients was higher among patients suffering from autoimmune or inflammatory (Lussana and Rambaldi, 2017; Wang and Zuo, 2019). Moreover; it has been reported that the main source of inflammatory cytokines are neoplastic clone and its differentiated progeny. These cytokines are released in the tumor microenvironment and leads to cytokine storm within the bone marrow niche which subsequently induces to marrow fibrosis (Lussana and Rambaldi, 2017). The time from start of disease to transformation to MF is usually differed being shorter in PV than ET and according to JAK2 allele burden and CALR mutations as well as cooperation of myeloid genes (Ferrer-Marín etal., 2020).

miR-146a-5p has been described as a negative regulator in innate immune and inflammatory responses mediated by Toll like receptor 4. Signaling downstream of TLR4 predominantly activates NF-κB after sequential activation of intermediate targets namely IRAK1 and TRAF6 (Taganov et al., 2006), which drives immune responses like leukocyte recruitment and pro-inflammatory cytokine production, exacerbating any disease (Jin et al., 2011). Also; it is the unique miRNA related to immune and inflammatory response with polymorphisms (miRSNPs) that influence its expression levels: rs2431697 (~1 kb upstream of of miR146A gene) and rs2910164 (within miR146A gene) (Lofgren et al., 2012). Interestingly, these two miRSNPs have been related with inflammatory response associated with certain diseases (Ramkaran et al., 2014).

NF-κB has been proposed as a key mediator of inflammation-induced carcinogenesis (Grivennikov et al 2010, Karin and Greten, 2005). As in other malignancies, NF-κB activation is detected in MF (Chorzalska et al., 2012). miR-146a is a significant brake on autoimmunity, myeloproliferation, and cancer in mice ( Boldineta., 2011).

This study aimed to delineate the association between miR-146a (rs2431697) polymorphisms and certain inflammatory biomarkers and the hazards of myelofibrosis progression. 

## Materials and Methods

*Patients and Methods*

All patients diagnosed as MPN who presented at Mansoura University Oncology Center (MUOC) between January 2015 up to September 2020 were included in cohort prospective study. The study included 88 patients (median age 62 years; age range 45-76) (45 Male; 43 Female). MPN (40 PV; 27ET; 21MF). The diagnosis of MPN was carried out according to the 2016 World Health Organization (WHO) (Grinfeld et al 2018). The MPN diagnostic subtypes comprised MPN (40 PV; 27ET; 21MF) recruitment at Mansoura University Oncology Center. Ninety healthy controls matched for age and sex with negative past history of hematological disorders were included as controls. EDTA-anticoagulated bone marrow samples from MPN patients and peripheral blood from healthy donors were collected. The investigated persons characteristics were shown in Table1. The study was approved by Mansoura University IRB committee.

*DNA extraction and genotyping*

The SNP rs2431697 is situated in the intergenic region between the Pituitary tumor-transforming gene 1 (PTTG1) and miR146a genes. The Genomic DNA was extracted from the peripheral blood samples collected in EDTA test tubes using a DNA Purification Kit (Promega, Madison, WI, USA). miRNA-146a rs2910164 C>G polymorphism genotyping was analyzed. In brief, a 150 ng DNA sample was heated to 98°C and held for 5 minutes. The ligation reaction was carried out in an ABI 2720 thermal cycler. Then, a 48-plex fluorescence polymerase chain reaction (PCR) was conducted. In an ABI 3730XL sequencer, capillary electrophoresis was harnessed to analyze the PCR products. Gene Mapper 4.1 software (Applied Biosystems, Foster City, CA, USA) was used to read the information of the genotype. For quality control, different technicians genotyped 4% of the genomic DNA samples that were randomly selected. And, the results were in full accord with the findings of the first assays.

*RNA isolation, cDNA synthesis and quantitative real-time PCR*

Fresh peripheral blood samples were collected in tubes containing EDTA. Lysis of red cells were done immediately and about 1 μg total RNA from each blood samples was extracted using Trizol (Invitrogen) and after that complementary DNA (cDNA) was synthesized from total RNA using Superscript II reverse transcriptase (Invitrogen) and oligo (dT) primers according to manufacturer instructions.

PCR was performed with the following thermo-cycling conditions: An initial 5 min at 95°C, followed by 40 cycles of 95°C for 30 sec, 55°C for 30 sec and 72°C for 30 sec. 

The real-time PCR system contained 5 μl of 2 × SYBR Green Real-time PCR Master Mix, 0.8 μl of the forward and reverse primers and 1 μl of cDNA, in a final volume of 10 μl. The relative mRNA expression of NLRP3, NF-κB1, IL-1β, was determined by ABI Prism 7500 real-time PCR system (Applied Biosystems, Foster City, CA, USA). Primers sequences used for reverse transcription-quantitative polymerase chain reaction analysis (15). NF-κB1 Forward (F) 5’-TCCAGACCAACAACAACCCC-3`, Reverse (R) GATCTTCTCGGCAGTGT-3`; for NLRP3 F. 5’-CAGACTTCTGTGTGTGGGACTGA-3`, R. 5’-TCCTGACAACATGCTGATGTGA-3`; For GAPDH F, 5’-GCTCTCTGCTCCTCCTGTTC; R. GTTGACTCCGACCTTCACCT-3`; for miR-146a F. 5’-TGAGAACTGAATTCCATGGGTT-3`, R. 5’-GCTGTCAACGATACGCTACGTAACG; for U6 F. 5’- GCTTCGGCAGCACATATACTAAAAT-3`, R. 5’-CGCTTCACGAATTTGCGTGTCAT-3`. 

All experiments were done in duplicate. The number of GAPDH transcript was used as an internal control for IL-1β; NF-κB1; NLRP3 and U6 was used as internal control for miR-146a. Transcript levels were expressed as arbitrary units and were calculated using the comparative threshold cycle method. Melting curve analysis was applied in all the PCR products.

*Statistical analyses*

Continuous variables are presented as the mean ± SD and categorical variables are presented as percentages. Comparisons of categorical variables between groups were carried out using the *χ*^2^ test for tables, while numerical variables were compared using the two-tailed Student’s t test or Mann–Whitney U test, where appropriate. Shapiro–Wilk test and Leveneʼs test were used to check normal distribution and homoscedasticity assumptions, respectively. The impact of TT on Transformation to MF is assessed by was evaluated by Cox regression.

**Table 1:** Main Patients Data of PV and ET Patients at the Time of Diagnosis

Parameters	Control	PV	ET	MF (Post PV and ET)	P
	(n=90)	(n=40)	(n=27)	(n=21)	Value
Age/years Median (Range)	61(45-76)	56(45-70)	53(45-65)	62(52-72)	≤0.001
Sex					
Male, no (%)	46(51.1)	19(47.5)	14(46.4)	12(5.5)	0.9
Female, no (%)	44(48.9)	21(52.5)	13(53.6)	9(45.5)	
Hemoglobin, g/l	13.5 ± 1.2	17.5 ± 1.7	14.1 ± 1.5	16.2 ± 4.5	≤0.001
WBC count, ×10^9^/l	8.2 ± 2.2	16.5 ± 4.4	8.7 ± 3.2	9.23 ± 1.8	≤0.001
Platelet count, ×10^9^/l	230 ± 32	597 ± 288	816 ± 289	420 ± 42	≤0.001
JAK2 mutated, n (%)	-	40 (100%)	14 (52%)	16(76%)	≤0.001
CALR mutated, n (%)	-	22 (55%)	23 (51.8%)		
MPL mutated, n (%)	-	1(2.5%)	2 (7.4%)		
Follow up time (years)		6	6		

**Table 2:** miR-146a rs2431697 Polymorphisms in Different Studied Group

Genotypes	Control (n= 90)	PV (n=40)	ET (n=27)	MF (n= 21)
CC	20 (22.2)	10 (25.0)	7 (25.9)	5 (23.8)
TC	59 (65.6)	20 (50.0)	12 (44.4)	5 (23.8)
TT	11 (12.2)	10 (25,0)	8 (29.6)	11 (52.4)
P value	P1 >0.05	P2 >0.05	P3 >0.05	P4 ≤0.001

P1, genotypesin Controlvs PV; P2, genotypes in controlvs ET; P3, genotypes in PV vs ET; P4, genotypes in MF vs PV and ET

**Table 3:** Genes Expressions of Inflammatory Mediators in All Studied Persons

	Control (n= 90)	PV (n=40)	ET (n=27)	MF (n= 21)	P value
	Median (min-max)	Median (min-max)	Median (min-max)	Median (min-max)	
IL-1β	0.35 (0.1-0.95)	1.3 (0.2-2.4)	1.2 (0.2-2.3)	1.7 (0.2-2.6)	0.002
NF-κB1	0.4 (0.2-1.2)	0.7 (0.2-2.1)	0.7 (0.2-1.4)	0.9 (0.2-1.9)	≤0.001
NLRP3	0.44 (0.1-1.33)	1.2 (0.2-3.4)	0.6 (0.5-2.4)	0.9 (0.2-1.9)	≤0.001
miR-146a	1.2 (0.77-2.23)	0.55 (0.45- 2.44)	0.48 (0.34-1.5)	0.22 (0.0 -0.92)	0.001

**Table 4:** Impact of miR-146a Genotypes on Inflammatory Genes Expression in All Studied Groups

	Control	PV	ET	MF
	Median (min-max)	Median (min-max)	Median (min-max)	Median (min-max)
IL-1β				
CC	0.18 (0.1-0.25)	0.47 (0.24-1.1)	0.56 (0.2-0.95)	0.4 (0.2-0.6)
TC	0.6 (0.22-0.87)	1.4 (0.45-1.95)	1.1 (0.2-1.4)	1.6 (0.6-1.7)
TT	0.77 (0.54-0.95)	2.1 (1.4-2.4)	2.0 (1.3-2.3)	2.1 (1.6-2.6)
P value	≤0.001	≤0.001	≤0.001	≤0.001
NF-κB1				
CC	0.39 (0.21-0.95)	0.32 (0.22-0.7)	0.34 (0.2-0.7)	0.19 (0.15-0.44)
TC	0.65 (0.23-0.92)	0.8 (0.2-2.1)	0.65 (0.2-0.9)	0.67 (0.2-0.9)
TT	0.94 (0.43-1.2)	1.2 (0.8-1.9)	1.2 (1.0-1.4)	1.4 (0.9-1.9)
P value	≤0.001	≤0.001	≤0.001	≤0.001
NLRP3				
CC	0.13 (0.1-0.23)	0.58 (0.23-0.66)	0.7 (0.5-1.1)	0.7 (0.5-1.0)
TC	0.55 (0.12-0.88)	1.4 (0.7-2.2)	1.3 (1.0-1.8)	1.8 (0.9-1.9)
TT	0.8 (0.22-1.33)	2.8 (2.0-3.4)	2.1 (1.9-2.4)	2.9 (2.4-3.2)
P value	≤0.001	≤0.001	≤0.001	≤0.001

**Table 5:** Correlation between miR146a and Inflammatory Genes Expression in Total MPN Group of Patients

	IL-1β	NF-κB1	NLRP3
*miRNA146a*	r= - 0.632 P<0.01	r= - 0.574 P<0.01	r= - 0.483 P<0.01

**Table 6:** Association between miR-146a rs2431697 Polymorphism Genotype and PV and ET Transformation to MF

Genotypes	PV (n=40)	ET (n=27)	MF (n=21)	MF vs.PV	MF vs.ET
	N (%)	N (%)	N (%)	OR (95% CI)	OR (95% CI)
CC	10 (25.0)	7 (25.9)	5 (23.8)	r (1)	r (1)
TC	25 (62.5)	12 (44.5)	5 (23.8)	0.3 (0.1-1.4)	0.5 (0.1-2.1)
TT	5 (12.5)	8 (29.6)	11 (52.4)	3.5 (0.8-16.7)	1.6 (0.4-7.0)

OR, Odds ration; r, reference category; CI,Confidence interval

## Results

*Gene frequency of miR-146a rs2431697 polymorphism in different studied groups*

The frequency of miR-146a rs2431697 genotypes in PV and ET did not significantly different (P>0.05). The T allele of rs2431697 is more frequent in MF case group as compared with the control group. The genotype TT is more frequent in MF group as compared to both PV; ET and control group (P=0.001) (Table 2).

*Expression of genes involved in NLRP3 inflammasome signaling were increased in MPN*

The expression of IL-1β; NF-κB1 and NLRP3 which are intracellular mediators are elevated in MPN subgroups as compared to controls (IL-1β; P=0.002; NF-κB1 P=≤0.001; NF-κB1 P=≤0.001; NLRP3 P=0.001). The highest levels were detected in MF group (Table 3).

*Association of the miR-146a rs2431697 SNP with miR-146a; IL-1β; NF-κB1 and NLRP3 genes expression*

It is evident that TT genotype is associated with lowest levels of IL-1β; NF-κB1 and NLRP3 genes expression in all studied MPN subgroups as compared to TC and CC genotypes (P<0.001 for all) (Table 4).

*Correlation studies between T allele of rs2431697 and inflammatory genes expression in MPN subgroups*

Correlation studies revealed that there is significant positive correlation between the 3 parameters in PV (IL-1β; NF-κB1 and NLRP3); ET (IL-1β; NF-κB1 and NLRP3) and MF (IL-1β; NF-κB1 and NLRP3) and significant negative correlation between T allele and miR-146a (Table 4).

*Correlation between mRNA146a expression and inflammatory mediators in total MPN group*

The statistical analysis in Table 5 revealed that there is negative significant correlation between mRNA146a expression and IL-1β; NF-κB1 and NLRP3.

*Odds ratio of miR-146a rs2431697 polymorphism genotype on PV and ET transformation to MF*

The TT genotype of miR-146a rs2431697 polymorphism was associated with the risk of transformation of PV and ET to MF with a OR: 3.5(CI: 0.8-16.7) in PV MPN subgroup and OR: 1.6(CI: 0.4-7.0) in ET and MPN subgroup respectively (Table 6).

## Discussion

The cardinal role of inflammatory process in the pathogenesis of MPN had emerged in many studies. Ferrer-Marín et al. (2020) and Masselli et al.(2019) have recently identified the 2518 A/G polymorphism in MCP-1, the main chemotactic factor for monocyte migration to sites of inflammation, as a potential genetic predisposition factor for secondary MF (Masselli et al., 2019).

The relationship between miR-146a and inflammatory process that is associated with MPN has been demonstrated in many previous reports. This reports stated that miR-146a modify the inflammatory process through accentuating NF-κB signaling (Taganov et al., 2006 and Guglielmelli etal., 2007). Likewise; symptoms of chronic inflammation that was described in MPN patients was attributed to increased inflammatory mediators in the serum of those patients. However; the relation between these inflammatory markers and progression to MF is not fully elucidated.

Our results showed that the rs2431697 TT genotype is frequently detected in MF subtypes of MPN patients as compared to controls. Similar finding was reported by Zhou et al., (2020). Lofgren et al., (2012) demonstrated that SNPs rs2431697 modify miR-146a expression levels. 

The expression of inflammatory mediators including NLRP3; IL-1β; NF-κB1 in PV; ET and MF were significantly elevated as compared to control group. Meanwhile the expression of these markers was significantly higher in MF group as compared to both PV and ET groups. These findings are similar to that reported by Zhou et al (2020) who found that inflammasome-related genes (NLRP3, NF-κB1, IL-1β) were highly expressed in BM cells from MPN patients and the increased expression was associated with JAK2V617F mutation, white blood cell counts and splenomegaly and suggested that these findings point out for novel biomarkers which may be a suggested target for MPN control. 

The association between rs2431697 TT genotype and inflammatory mediators’ expression has been evaluated in the current study. Our results revealed that the TT genotypes are associated with high expression of inflammatory-related genes namely NLRP3, NF-κB1, and IL-1β. These findings are in parallel with that reported in a previous recent study (Ferrer-Marín etal., 2020). 

Importantly, our findings suggest that the rs2431697 TT genotype not only increases susceptibility to secondary MF, but may also be considered a marker for early progression. These findings strongly suggest that the rs2431697 genotype represents a new prognostic factor for MPN, and may point to the development of new therapeutic strategies based on the use of novel agents such as miRNA mimics.

The limitation of this study is small sample size of our cohort of MPN patients. Large scale study is recommended to validate our findings.

In conclusion our findings demonstrated the impact of miR-146a polymorphism on its expression and subsequently the progression of MPN to MF. This is through down regulation of miR-146a expression and up regulation of the genes (NLRP3, NF-κB1, IL-1β) that control inflammations. Targeting NLRP3, NF-κB1, IL-1β genes might hinder MF progression in MPN patients.

## Author Contribution Statement

Salah Aref: Conception and study design and Manuscript revision; Doaa Atia: Laboratory work; Interpretation and analysis of data; Ahmed Al Tantawy: Preparation of the manuscript and Revision for Important intellectual; Mohamed Al Boghdady: Clinical assessment of Patients; Enas Gouda: Laboratory work; Interpretation and analysis of data.
